# Association of major dietary patterns and different metabolic phenotypes: a population-based study of northwestern Iran

**DOI:** 10.1186/s12902-019-0455-3

**Published:** 2019-12-03

**Authors:** Leila Nikniaz, Mahdieh Abbasalizad Farhangi, Jafar Sadegh Tabrizi, Zeinab Nikniaz

**Affiliations:** 1 Tabriz Health Services Management Research Center, Tabriz University of Medical Sciences, Tabriz, Iran; 2 Drug Applied Research Center, Tabriz University of Medical Sciences, Tabriz, Iran; 3 Tabriz Health Services Management Research Center, Tabriz University of Medical Sciences, Tabriz, Iran; 4 Liver and Gastrointestinal Diseases Research Center, Tabriz University of Medical Sciences, Tabriz, Iran

**Keywords:** Dietary pattern, Cardiometabolic profile, Metabolically healthy obese

## Abstract

**Background:**

Finding the relationship between the major dietary patterns and cardiometabolic phenotypes could be used for planning prevention programs based on the cultural and dietary habits to prevent transient from a metabolically healthy state to an unhealthy state. So, we aimed to assess the association between dietary patterns and cardiometabolic phenotypes in the northwestern population of Iran**.**

**Method:**

In the present cross-sectional and population-based study, 504 adults sampled by cluster sampling in East-Azerbaijan, Iran. Factor analysis was used for determining the dietary pattern. Metabolic phenotypes were determined according to body mass index (BMI) cut–off point (25 kg/m^2^), and the presence of the metabolic syndrome. The independent sample t-test, one-way ANOVA, chi-square, and multinomial regression were used for statistical analysis.

**Results:**

In both adjusted (OR: 2.24, 95% CI: 1.17, 4.31) and unadjusted models (OR: 3.14, 95% CI: 1.54, 5.42), the last tertile of the animal dietary pattern was associated with metabolically healthy obese (MHO) phenotypes. After adjusting, the last tertile of the animal dietary pattern was significantly associated with an increase of metabolically unhealthy obese (MUHO) phenotype (OR: 2.61, 95% CI: 1.18, 5.76).

**Conclusion:**

In the present study, the animal dietary pattern was associated with MHO and MUHO phenotypes. It is suggested that some measures should be taken to strengthen nutrition education for the population and advocate a balanced diet to improve the condition.

## Background

Obesity and over-weight are public health problems and they are the world’s fifth causes of mortality [1, 2]. The obesity rate has tripled during the last two decades [3]. In Iran, the total rate of obesity was reported as 12.3% in a recent meta-analysis study, which was calculated as 21.7% for people above 18 and as 6.5% for people less than 18 years of age. According to the recent world health organization (WHO) report (2010), more than half of the Iranian adult population are overweight or obese [4]. Obesity is shown to be associated with dyslipidemia, hyperglycemia, insulin resistance and hypertension [5]. However, currently, it is proposed that not all obese individuals are at high risk of developing these complications [6]. So, from the clinical point of view, it is important to identify factors that predispose some obese individuals to develop cardiovascular risk factors. Among all other reasons, dietary behaviors are one of the major determinants of developing obesity-related complications.

A cohort study conducted in the North of The Netherlands compared the dietary pattern of metabolically healthy and unhealthy obese people and showed that the ‘bread, potatoes and sweet snacks’ dietary pattern counteracted healthy metabolic phenotype in obese women [7]. Considering that the metabolically healthy obese (MHO) population was more likely to transit into the metabolically unhealthy obese (MUHO) over time [8, 9], finding the relationship between the major dietary patterns and cardiometabolic phenotypes could be used for planning prevention programs based on the cultural and dietary habits of this region. So, the present study aimed to examine the association between dietary patterns and cardiometabolic phenotypes in Tabriz-Iran.

## Method

This study used the cross-sectional data of the major lifestyle promotion project (LPP) conducted in East-Azerbaijan, Iran in 2015. A detailed sampling method is described in our previous study [10, 11]. The sampling frame was defined using the postal code. Probability proportional to size multistage stratified cluster sampling was used as a sampling method. For the aim of the present study, 550 participants from 150 clusters were joined. One adult was randomly selected from each household. Forty-six participants were excluded due to incomplete information and the final analysis was done on 504 participants.

### Ethics, consent, and permissions

The approval was obtained from the Ethics committee of Tabriz University of Medical Sciences (registration number: 1394.383). Moreover, written consent was obtained from all participants before participating in the study.

### Measurements

For biochemical measurements, 10 ml of fasting blood sample (being at least 10-h fasting) was collected. Lipid profile [High-Density Lipoprotein-Cholesterol (HDL-C) and triglycerides (TG)] and serum glucose were measured using enzymatic colorimetric method (Pars Azmoon commercial kit, Tehran, Iran). A standard manual sphygmomanometer was used for measuring blood pressure in a sitting position. The anthropometric tape was used for measuring waist circumference (WC) according to the standard method [12]. Body mass index (BMI), was calculated by dividing the body weight in Kg (measured with a Seca scale) by the square of the height in meter.

### Definition of metabolic syndrome (MetS) and cardiometabolic phenotypes

Metabolic syndrome was defined according to the adult Panel III (ATP III) criteria and if a participant met three or more of the following criteria, he/she was considered as having MetS: Abdominal obesity (WC ≥102 cm in men and ≥ 88 cm in women); hypertriglyceridemia (TG ≥ 150 mg/dl or on drug treatment for hypertriglyceridemia); hypo-high-density lipoprotein cholesterolemia (HDL-C < 40 mg/dl in men and < 50 mg/dl in women or on drug treatment for hypo-HDL- cholesterolemia); hypertension (systolic blood pressure ≥ 130 mmHg or diastolic blood pressure ≥ 85 mmHg or using antihypertensive drug) and high fasting glucose (fasting glucose ≥100 mg/dl or on drug treatment for hyperglycemia). Participants were categorized into four cardiometabolic phenotypes according to their BMI and the MetS status. These phenotypes include MHL: metabolically healthy lean (individuals with BMI lower than 25 kg/m^2^ and absence of metabolic syndrome); MUHL: metabolically unhealthy lean (individuals with BMI < 25 kg/m^2^ and presence of metabolic syndrome); MHO: metabolically healthy obese (individuals with BMI > 25 kg/m^2^ and absence of metabolic syndrome); and MUHO: metabolically unhealthy obese (individuals with BMI > 25 kg/m^2^ and presence of metabolic syndrome).

### Dietary assessment

An 80-item quantitative food frequency questionnaire (FFQ) was used for the evaluation of dietary factors. This questionnaire was developed and validated for the LPP study [13] and completed through face-to-face interviews by expert dietitians.

The frequency and amount of food items consumption were reported over the last year on a daily, weekly or monthly basis and the reported amounts were converted into daily consumption. During the face to face interview, household scales displayed in colored photographs were used to help the respondent’s memory. According to nutrient content, each food item was assigned to one of the defined food groups. Twenty three food groups were defined according to nutrient content including red meat, organ meat, fish, poultry, nuts, eggs, dairy products, tea, coffee, fruits, fruit juices, vegetables, legumes, whole grains, refined grains, fast foods, dry fruits, sweets and desserts, animal fats, liquid vegetable oil, soft drinks, salt and pickle. The detail of the food items included in each food group is presented in Table 1 in our previous article [14]. For deriving dietary patterns, the principal component analysis with varimax rotation was applied and the factors were retained based on their natural interpretation, eigenvalues> 1 and scree plots [15]. The factor scores were calculated for each dietary pattern. The factors were named by interpretation of the data and considering the prior literature.

**Table 1:** Baseline characteristics of participants

Variables	Total (*n* = 504)	Males (*n* = 208)	Females (*n* = 296)	*p*-value*
Age (mean ± SD)	42.40 ± 12.38	43.58 ± 13.57	41.64 ± 11.40	0.07
Education level, n (%)				0.001
illiterate	74 (14.68)	15 (7.21)	59 (19.93)	
≤ High school/ diploma	361 (71.62)	157 (75.48)	204 (68.91)	
≥ college degree	69 (13.69)	36 (17.30)	33 (11.14)	
Marital status, n (%)				0.001
Single	45 (8.92)	30 (14.42)	15 (5.06)	
Married	459 (91.07)	178 (85.57)	281 (94.59)	
Current smoker, n (%)	58 (11.50)	55 (26.44)	3 (1.01)	< 0.001
WC (cm)	93.08 ± 12.88	94.43 ± 13.28	92.12 ± 12.53	0.51
FBS (mg/dl)	89.71 ± 30.39	90.50 ± 28.33	89.15 ± 31.85	0.72
SBP (mmHg)	120.57 ± 12.68	121.54 ± 18.24	119.89 ± 31.85	0.59
DBP (mmHg)	78.12 ± 11.63	78.52 ± 12.23	77.84 ± 11.19	0.69
TG (mg/dl)	160.46 ± 103.41	182.30 ± 119.77	145.16 ± 87.85	< 0.001
HDL-C (mg/dl)	43.70 ± 9.69	39.68 ± 8.29	46.55 ± 9.61	0.004
Cardio metabolic phenotype n (%)				
MHL	135 (26.78)	69 (31.17)	66 (22.29)	0.79
MUHL	17 (3.37)	7 (3.36)	10 (3.37)	0.46
MHO	204 (40.47)	83 (39.90)	121 (40.87)	0.008
MUHO	148 (29.36)	49 (23.55)	99 (33.44)	< 0.001

MHL: Metabolically Healthy Lean; MUHL: Metabolically unhealthy lean; MHO: Metabolically healthy obese; MUHO: Metabolically unhealthy obese; WC: waist circumference; FBS: fasting blood sugar; SBP: systolic blood pressure; DBP: diastolic blood pressure; TG: triglyceride; HDL-C: high density lipoprotein-cholesterol

*continuous variables were compared by independent t-test and categorical variables were compared using chi-square and fisher exact test

Covariates for regression analyses were age, smoking status, educational level, physical activity, and dietary intake. The socio-demographic characteristics and smoking status were collected through questionnaires. For evaluating the physical activity level, the international physical activity questionnaire-short form (IPAQ-SF) was used [16].

### Statistical analysis

SPSS V18 statistical computer software was used for all statistical analyses. Major dietary patterns were identified using principal component analysis. Factor scores were categorized in tertiles according to the distribution of participants with each pattern. The significant differences in continuous variables were determined using an independent sample t-test. One-way ANOVA and χ2 and Fisher exact tests were used to examine the differences in metabolic syndrome components and physical activity level and smoking status across tertiles. Multinomial regression in different models (unadjusted model and adjusted model which adjusted for age, sex, BMI, physical activity, smoking status, and energy intake) was used to determine the association between dietary patterns (independent variable) and cardiometabolic phenotypes (dependent variable). Significance level was set at *p*-value< 0.05.

## Results

Using factor analysis, three major dietary patterns were identified: animal dietary pattern (including red meat, organ meat, poultry, fish, nuts, dry fruits), healthy dietary pattern (including dairy products, fruits, fruit juices, vegetables, legumes, coffee and tea) and Western dietary pattern (including refined grain, fast foods, sweets and desserts, animal fats, vegetable oils, soft drinks, egg and salt; whole grains negatively loaded on this pattern). These dietary patterns explained 23.53% of the whole variance. Detail of the factor loading matrix is given in our previous article [14].

The descriptive characteristics of participants are shown in Table 1. The mean age of the study population was 42.40 ± 12.38 years, 14.68% of them were illiterate and about 91.07% of them were married. About 26.78% of the participants presented the MHL phenotype. There were significant differences between males and females regarding education level (*P* = 0.001), marital (P = 0.001) and smoking (*P* < 0.001) status. As apparent from Table 1, the MHO and MUHO phenotypes were more prevalent in females compared to males.

As presented in Table 2, in both sex groups, there were no significant differences in mean energy intake and physical activity levels between different cardiometabolic groups. However, in the case of BMI and WC, ANOVA statistical test showed the significant differences between different groups of cardiometabolic phenotypes. Among different phenotypes, participants presented the MHL phenotype had the lowest mean BMI and WC and participants presented MUHO phenotype had the highest mean BMI and WC. Among both sex groups and also in both lean and obese participants, BMI and WC in metabolically healthy subjects were lower than metabolically unhealthy subjects.

**Table 2:** Anthropometric measurements, dietary intake and physical activity status across different cardiometabolic phenotypes

Variables	MHL (*n* = 69)	MUHL (*n* = 7)	MHO (*n* = 83)	MUHO (*n* = 49)	*p*-value*
Men (n = 208)					
Physical activity n (%)					
No PA	22 (31.88)	2 (28.57)	27 (32.53)	22 (44.89)	0.56
Low PA	24 (34.78)	1 (14.28)	26 (31.32)	12 (24.48)	
High PA	23 (33.33)	4 (57.14)	30 (36.14)	15 (30.61)	
Energy (Kcal/day)	3862 ± 1520	4388 ± 946.6	3753 ± 3177	3741 ± 1520	0.47
Carbohydrate (g/day)	216.65 ± 65.53	304.42 ± 146.74	218.35 ± 19.95	208.41 ± 81.11	0.11
Protein (g/day)	218.18 ± 94.56	228.48 ± 61.62	199.02 ± 86.95	175.21 ± 68.46	0.11
Fat (g/day)	269.89 ± 118.18	264.72 ± 89.85	246.38 ± 112.87	221.23 ± 89.87	0.23
WC (cm)	82.93 ± 13.43	89.57 ± 12.44	97.20 ± 8.81	103.28 ± 9.95	< 0.001
BMI (Kg/m^2^)	22.23 ± 2.31	23.92 ± 0.83	28.23 ± 3.15	30.51 ± 4.06	< 0.001
Women (*n* = 296)	MHL (*n* = 66)	MUHL (*n = *10)	MHO (*n* = 121)	MUHO (*n* = 99)	*p*-value*
Physical activity					
No PA	26 (39.39)	2 (20)	45 (37.19)	28 (28.28)	0.08
Low PA	13 (19.69)	2 (20)	41 (33.88)	38 (38.38)	
High PA	27 (40.90)	6 (60)	35 (28.92)	33 (33.33)	
Energy (Kcal/day)	2818 ± 1318	2837 ± 757	3163 ± 1560	3272 ± 1326	0.31
Carbohydrate (g/day)	179.23 ± 70.46	175.56 ± 48.49	208.29 ± 84.40	217.30 ± 93.38	0.63
Protein (g/day)	141.05 ± 74.79	132.93 ± 33.56	159.85 ± 82.81	156.01 ± 74.75	0.45
Fat (g/day)	175.77 ± 98.11	182.43 ± 58.18	201.75 ± 106.56	200.44 ± 100.17	0.46
WC (cm)	79.48 ± 10.48	91.00 ± 6.56	91.56 ± 10.35	100.79 ± 9.02	< 0.001
BMI (Kg/m^2^)	22.21 ± 2.03	23.95 ± 0.85	29.39 ± 3.45	31.85 ± 4.38	< 0.001

MHL: Metabolically Healthy Lean; MUHL: Metabolically unhealthy lean; MHO: Metabolically healthy obese; MUHO: Metabolically unhealthy obese; BMI: Body Mass Index; WC: waist circumference; PA: physical activity

*continuous variables were compared by one way ANOVA and categorical variables were compared using chi-square and fisher exact test

Correlation between cardiometabolic phenotypes and different dietary patterns are presented in Table 3. In both unadjusted (OR: 2.24, 95% CI: 1.17, 4.31) and adjusted models (OR: 3.14, 95% CI: 1.54, 5.42), participants with high adherence to the animal dietary pattern compared to those with lower consumption of this pattern were more likely to present the MHO phenotype than MHL phenotype. Moreover, in unadjusted model, there was no significant association between animal dietary pattern and MUHO phenotype (OR: 1.57, 95% CI: 1.18, 5.76), however, in adjusted model, we showed that participants with higher consumption of animal dietary pattern compared to those with lower consumption, were more likely to present MUHO phenotype than MHL phenotype (OR: 2.61, 95% CI: 1.18, 5.76). No significant association was found between two other dietary patterns (healthy and western) and the odds of cardiometabolic phenotypes in our population.

**Table 3:** Correlation between cardiometabolic phenotypes and different dietary pattern

Dietary patterns	MUHL OR (95% CI)		MHO OR (95% CI)		MUHO OR (95% CI)	
	Unadjusted	Adjusted*	Unadjusted	Adjusted*	Unadjusted	Adjusted*
Animal dietary pattern						
1st tertile	1	1	1	1	1	1
2nd tertile	1.09 (0.31, 3.78)	1.51 (0.30, 7.61)	1.29 (0.69, 2.40)	1.53 (0.78, 3.11)	0.85 (0.44, 1.75)	1.20 (0.56, 2.54)
3rd tertile	0.76 (0.17,3.41))	1.67 (0.43, 6.47)	2.24 (1.17, 4.31)	3.14 (1.54, 6.42)	1.57 (0.79, 3.12)	2.61 (1.18, 5.76)
Healthy dietary pattern						
1st tertile	1	1	1	1	1	1
2nd tertile	0.44 (0.11, 1.64)	0.56 (0.13, 2.38)	1.01 (0.53, 1.91)	1.23 (0.69, 2.44)	1.44 (0.74, 2.80)	2.11 (0.98, 4.51)
3rd tertile	0.47 (0.11, 1.99)	0.42 (0.09, 1.97)	1.51 (0.80, 2.85)	1.35 (0.68, 2.69)	1.21 (0.60,2.43)	1.18 (0.53, 2.59)
Western dietary pattern						
1st tertile	1	1	1	1	1	1
2nd tertile	1.82 (0.32, 10.20)	3.36 (0.81, 20.78)	0.55 (0.29, 1.05)	0.71 (0.35, 1.41)	0.46 (0.23, 0.91)	0.71 (0.33, 1.53)
3rd tertile	3.79 (0.72, 19.88)	4.76 (0.81, 21.81)	0.92 (0.51, 1.91)	1.22 (0.59, 2.52)	0.71 (0.35,1.42)	1.02 (0.45, 2.29)

Metabolically Healthy Lean (MHL) was considered as reference group

MUHL: Metabolically Unhealthy Lean; MHO: Metabolically Healthy Obese; MUHO: Metabolically Unhealthy Obese. OR: odds ratio; CI: confidence interval

*Adjusted for age, sex, BMI, Physical activity, smoking status and energy intake

## Discussion

To the best of our knowledge, for the first time in Iran, this study evaluated the association between dietary patterns and cardiometabolic phenotypes. Considering the results, the prevalence of MHL, MUHL, MHO, and MUHO was 26.78, 3.37, 40.47, and 29.36% respectively; implying that more than half of the obese population in the present study was metabolically healthy. There is a discrepancy between the prevalence of cardiometabolic phenotypes in different populations [17–21] that may be due to the differences in metabolic phenotype definition in different studies and also differences in dietary and lifestyle habits. Consistent with the results of other studies [19], there was a significant difference in mean BMI and waist circumference between metabolically unhealthy and healthy individuals. It may be suggested that excess visceral fat tissue may explain the presence of metabolically unhealthy characteristics. In the present study, we observed that in both sexes, there were no significant differences in mean energy intake and physical activity levels between different cardiometabolic groups. Unlike our expectation, we did not observe lower mean energy intake in lean participants that may be due to the cross-sectional design of the study or the limitations of FFQ in determining absolute intakes.

In the present study, the mean BMI and WC were significantly higher in individuals presented MUHO phenotype compared with participants presented MHO phenotype. Moreover, the animal dietary pattern was associated with MHO and MUHO phenotypes. So, these findings may suggest that although animal dietary pattern could not prevent obesity, in subjects with lower waist circumference and lower BMI (overweight subjects) it may reduce the metabolic-abnormalities associated with obesity. This positive effect may be due to the high consumption of nuts, dry fruits, and fish in this dietary pattern. Previously, Carughi et al., in a review article concluded that consumption of nuts and dry fruits prevent metabolic abnormalities and chronic diseases [22]. In another systematic review, TØRRIS and colleagues have suggested that fish consumption may prevent or improve metabolic health and have a positive role in MetS prevention [23].

In obese participants, we showed that animal dietary pattern could not prevent obesity and metabolic abnormalities. In addition to healthy foods, red meat and organ meats were also loaded on this dietary pattern. In a previous cross-sectional study in Iran, Azadbakht and Esmailzadeh showed that increased red meat consumption is associated with a greater risk of metabolic syndrome. Moreover, the association between dietary pattern and cardiometabolic phenotypes may partly be explained by gene-environment interaction [24]. In animal studies, it has been shown that mutations in Brd2 gene could prevent type 2 diabetes in mouse [25].

Contrary to our expectation, there was not any relationship between healthy dietary pattern and metabolically healthy phenotypes. In the present study, dairy product consumption was loaded on the healthy dietary pattern. The Iranian population is mostly using full-fat dairy products [26] that could be considered as a contributing factor to metabolic syndrome [27].

Present study limitations were including the inability to make causal inferences due to its cross-sectional design. Moreover, for evaluating dietary intake, the FFQ was used that has some limitations including inaccurate describing of portion size and its dependence on memory. These limitations could also apply to the dietary pattern method. Meanwhile, a trained health professional administered FFQ which increases the validity of data. A large sample size, using an appropriate method of sampling, and also controlling for a large number of potential confounders such as dietary intake and physical activity could be considered as the strength of the present study. Because of the method of sampling and also sample size, the study’s participants could be considered as representatives of East-Azerbaijan province population.

## Conclusion

In conclusion, the results of the present study showed that the prevalence of MUHL and MHO was 3.2 and 38.4%. Moreover, according to the results of multinomial regression analysis, the animal dietary pattern was associated with both MHO and MUHO phenotypes. From the research point of view, these findings showed that the knowledge about the association between dietary pattern and cardiometabolic phenotype is limited and because of the uniqueness of the dietary pattern and also some special food items in each region, the study about this association in each region is necessary. Moreover, in future studies, consideration of the genetic variation of participants could more precisely define the association between dietary patterns and cardiometabolic phenotypes.

## Data Availability

The datasets supporting the conclusions of this research are included within the article.

## Abbreviations

BMI: Body Mass Index
CI: confidence interval
DBP: diastolic blood pressure
FBS: fasting blood sugar
HDL-C: high density lipoprotein-cholesterol
MHL: Metabolically Healthy Lean
MHO: Metabolically healthy obese
MUHL: Metabolically unhealthy lean
MUHO: Metabolically unhealthy obese
OR: Odds ratio
PA: physical activity
SBP: systolic blood pressure
SD: standard deviation
TG: triglyceride
WC: waist circumference

## References

[1] Janghorbani M, Amini M, Willett WC, Gouya MM, Delavari A, Alikhani S, Mahdavi A (2007). First nationwide survey of prevalence of overweight, underweight, and abdominal obesity in Iranian adults. Obesity.

[2] Ayatollahi S, Ghoreshizadeh Z (2010). Prevalence of obesity and overweight among adults in Iran. Obes Rev.

[3] Hossain P, Kawar B, El Nahas M (2007). Obesity and diabetes in the developing world—a growing challenge. New Engl J Med.

[4] Organization WH: Global status report on noncommunicable diseases 2014: World Health Organization; 2014.

[5] Kim JJ, Li P, Huntley J, Chang JP, Arden KC, Olefsky JM (2009). FoxO1 haploinsufficiency protects against high-fat diet–induced insulin resistance with enhanced peroxisome proliferator–activated receptor γ activation in adipose tissue. Diabetes.

[6] Karelis AD, Faraj M, Bastard J-P, St-Pierre DH, Brochu M, Prud’homme D, Rabasa-Lhoret R (2005). The metabolically healthy but obese individual presents a favorable inflammation profile. J Clin Endocrinol Metab.

[7] Slagter SN, Corpeleijn E, Van Der Klauw MM, Sijtsma A, Swart-Busscher LG, Perenboom CW, De Vries JH, Feskens EJ, Wolffenbuttel BH, Kromhout D (2018). Dietary patterns and physical activity in the metabolically (un) healthy obese: the Dutch lifelines cohort study. Nutr J.

[8] Appleton SL, Seaborn CJ, Visvanathan R, Hill CL, Gill TK, Taylor AW, Adams RJ, Team NWAHS (2013). Diabetes and cardiovascular disease outcomes in the metabolically healthy obese phenotype. Diabetes Care.

[9] Soriguer Federico, Gutiérrez-Repiso Carolina, Rubio-Martín Elehazara, García-Fuentes Eduardo, Almaraz María Cruz, Colomo Natalia, Esteva de Antonio Isabel, de Adana María Soledad Ruiz, Chaves Felipe Javier, Morcillo Sonsoles, Valdés Sergio, Rojo-Martínez Gemma (2013). Metabolically Healthy but Obese, a Matter of Time? Findings From the Prospective Pizarra Study. The Journal of Clinical Endocrinology & Metabolism.

[10] Tabrizi JS, Farahbakhsh M, Bazargani HS, Nikniaz L (2016). Introducing the objectives, procedures and structure of lifestyle promotion project (LPP): phase I. DepHealth.

[11] Tabrizi JS, Farahbakhsh M, Sadeghi-Bazargani H, Nikniaz L (2018). Prevention and control of non-communicable diseases in Iranian population: life style promotion project phase II: study protocol. Iran J Public Health.

[12] World Health Organization: Waist circumference and waist-hip ratio: report of a WHO expert consultation, Geneva, 8–11 December 2008. In*.* Geneva, Switzerland; 2011.

[13] Nikniaz Leila, Tabrizi Jafarsadegh, Sadeghi-Bazargani Homayoun, Farahbakhsh Mostafa, Tahmasebi Sanaz, Noroozi Soheila (2017). Reliability and relative validity of short-food frequency questionnaire. British Food Journal.

[14] Nikniaz L, Nikniaz Z, Sadeghi-Bazargani H, Abdollahi HM, Farhangi MA. Association between major dietary patterns and metabolic syndrome components: a population-based study from north-west of Iran. Int J Diab Dev Countries. 2019:1–9.

[15] J-o K, Mueller CW (2000). Factor analysis: statistical methods and practical issues.

[16] IPAQ Research Committee: International physical activity questionnaire: Short last 7 days self-administered format. http://www.ipaqkise 2005.

[17] Goday A, Calvo E, Vázquez LA, Caveda E, Margallo T, Catalina-Romero C, Reviriego J (2016). Prevalence and clinical characteristics of metabolically healthy obese individuals and other obese/non-obese metabolic phenotypes in a working population: results from the Icaria study. BMC Public Health.

[18] Matta J, Nasreddine L, Jomaa L, Hwalla N, Mehio Sibai A, Czernichow S, Itani L, Naja F (2016). Metabolically healthy overweight and obesity is associated with higher adherence to a traditional dietary pattern: a cross-sectional study among adults in Lebanon. Nutrients.

[19] Popa S, Moţa M, Popa A, Moţa E, Serafinceanu C, Guja C, Catrinoiu D, Hâncu N, Lichiardopol R, Bala C (2016). Prevalence of overweight/obesity, abdominal obesity and metabolic syndrome and atypical cardiometabolic phenotypes in the adult Romanian population: PREDATORR study. J Endocrinol Investig.

[20] Pajunen P, Kotronen A, Korpi-Hyövälti E, Keinänen-Kiukaanniemi S, Oksa H, Niskanen L, Saaristo T, Saltevo JT, Sundvall J, Vanhala M (2011). Metabolically healthy and unhealthy obesity phenotypes in the general population: the FIN-D2D survey. BMC Public Health.

[21] Kaur J. A comprehensive review on metabolic syndrome. Cardiol Res Pract. 2014;2014. 10.1155/2014/943162 PMC3966331 24711954

[22] Carughi A, Feeney MJ, Kris-Etherton P, Fulgoni V, Kendall CW, Bulló M, Webb D (2016). Pairing nuts and dried fruit for cardiometabolic health. Nutr J.

[23] Tørris C, Molin M, Småstuen MC (2014). Fish consumption and its possible preventive role on the development and prevalence of metabolic syndrome-a systematic review. Diabetol Metab Syndr.

[24] Azadbakht L, Esmaillzadeh A (2009). Red meat intake is associated with metabolic syndrome and the plasma C-reactive protein concentration in women. J Nutr.

[25] Roberson LL, Aneni EC, Maziak W, Agatston A, Feldman T, Rouseff M, Tran T, Blaha MJ, Santos RD, Sposito A (2014). Beyond BMI: the “metabolically healthy obese” phenotype & its association with clinical/subclinical cardiovascular disease and all-cause mortality--a systematic review. BMC Public Health.

[26] Ghassemi H, Harrison G, Mohammad K (2002). An accelerated nutrition transition in Iran. Public Health Nutr.

[27] Esmaillzadeh A, Kimiagar M, Mehrabi Y, Azadbakht L, Hu FB, Willett WC (2007). Dietary patterns, insulin resistance, and prevalence of the metabolic syndrome in women. Am J Clin Nutr.

